# Development of membrane-insertable lipid scrambling peptides: A time-resolved small-angle neutron scattering study

**DOI:** 10.1063/4.0000045

**Published:** 2021-03-19

**Authors:** Hiroyuki Nakao, Yusuke Kimura, Ami Sakai, Keisuke Ikeda, Minoru Nakano

**Affiliations:** Department of Biointerface Chemistry, Faculty of Pharmaceutical Sciences, University of Toyama, 2630 Sugitani, Toyama 930-0194, Japan

## Abstract

Phospholipid transbilayer movement (flip-flop) in the plasma membrane is regulated by membrane proteins to maintain cell homeostasis and interact with other cells. The promotion of flip-flop by phospholipid scramblases causes the loss of membrane lipid asymmetry, which is involved in apoptosis, blood coagulation, and viral infection. Therefore, compounds that can artificially control flip-flop in the plasma membrane are of biological and medical interest. Here, we have developed lipid scrambling transmembrane peptides that can be inserted into the membrane. Time-resolved small-angle neutron scattering measurements revealed that the addition of peptides containing a glutamine residue at the center of the hydrophobic sequence to lipid vesicles induces the flip-flop of 1-palmitoyl-2-oleoyl-*sn*-glycero-3-phosphocholine. Peptides without the glutamine residue had no effect on the flip-flop. Because the glutamine-containing peptides exhibited scramblase activity in monomeric form, the polar glutamine residue would be exposed to the hydrocarbon region of the membrane, perturbing the membrane and promoting the lipid flip-flop. These scrambling peptides would be valuable tools to regulate lipid flip-flop in the plasma membrane.

## INTRODUCTION

The regulation of lipid transbilayer movement (flip-flop) plays an essential role in cellular functions and intercellular communication.^1^ In the plasma membrane (PM), several proteins have been identified which mediate the flip-flop of phospholipids. The steady-state asymmetric distribution of phospholipids is maintained by the energy-dependent unidirectional transport of phospholipids.^2^ In the case of apoptosis or blood coagulation, PM phospholipid scramblases are activated to promote the flip-flop, disrupting the asymmetry in an energy-independent manner.^3,4^ The loss of asymmetry is related to the engulfment of apoptotic cells by phagocytes and the binding of blood clotting factors to the membrane to form a complex.^5^ The deficiency of TMEM16F, a human PM scramblase, causes a bleeding disorder (Scott syndrome).^3^ Phospholipid flip-flop is also induced during viral infections.^6^ In addition, redistribution of phospholipids by scramblases regulates the activity of the mechanosensitive Ca^2+^ channel PIEZO1 in the PM.^7^ Artificially controlling phospholipid flip-flop in the PM, therefore, is of considerable biological and medical interest.

Amphiphilic peptides, such as magainin and melittin, can enhance phospholipid flip-flop in the bacterial cytoplasmic membrane by binding to the membrane and forming a toroidal pore.^8^ The toroidal pore structure is composed of several peptides and phospholipids with hydrophilic residues and a polar head group facing inward into the pore, respectively. As such, the lipids can easily move to the opposite leaflet through the pore without exposing their head groups to the hydrocarbon region of the membrane. Ohmann *et al.* recently developed membrane-spanning DNA nanostructures that form toroidal pores in the membrane and induce rapid flip-flop.^9^ The amphiphilic peptides exhibit antimicrobial activity through toroidal pore formation, which causes the leakage of intracellular contents and cell death. This cytotoxicity may prevent the utilization of compounds with the toroidal pore structure for the control of PM flip-flop in living cells. Therefore, it is desirable to develop compounds that can control PM flip-flop without forming a pore structure.

We previously developed lipid scrambling peptides with one or two strongly hydrophilic residues in their transmembrane region.^10–12^ These peptides act as scramblases in a monomeric form, indicating that they would not form a pore structure in the membrane. Because the peptides have lysine residues at both terminals of the sequence to anchor them to the membrane, the position of the lysine residues should be changed to make them membrane-insertable.

**FIG. 1.**
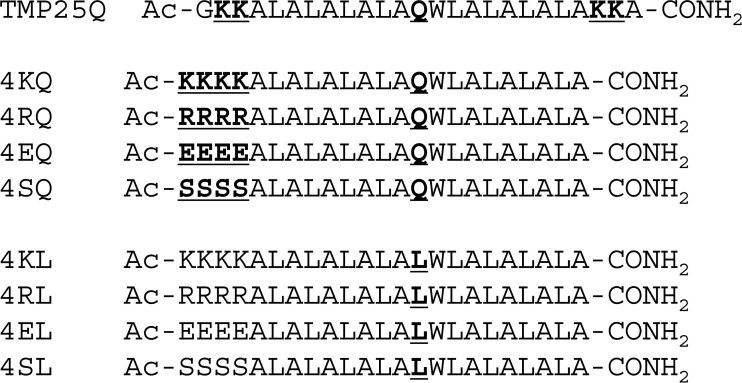
Amino acid sequences of TMP25Q, 4XQ, and 4XL peptides.

In the present study, we synthesized 4XQ peptides (X = K, R, E, and S) based on the sequence of a scrambling peptide, TMP25Q,^12^ where the glutamine residue is the active center for lipid scrambling (Fig. 1). The four X residues are located at the N-terminal to increase the solubility of the peptides and to allow the peptides to be inserted into the membrane from hydrophobic C-terminal regions. The scramblase activities of the peptides on naturally occurring lipids were investigated *in vitro* using time-resolved small-angle neutron scattering (SANS).^13,14^ The addition of 4XQ peptides to the membrane induced the flip-flop of 1-palmitoyl-2-oleoyl-*sn*-glycero-3-phosphocholine (POPC). The linear concentration dependence of the peptide activities suggests that the peptide monomers exhibit scramblase activity, which contrasts with the amphiphilic peptides that form toroidal pores. 4XQ peptides might be useful for the control of cellular functions and the treatment of scramblase-related diseases.

## EXPERIMENTAL METHODS

### Materials

POPC was purchased from NOF Corporation (Tokyo, Japan). 1-Palmitoyl-d_31_-2-oleoyl-*sn*-glycero-3-phosphocholine (*d*_31_-POPC) was purchased from Avanti Polar Lipids (Alabaster, AL, USA). D_2_O and methyl-β-cyclodextrin (MβCD) were obtained from Sigma-Aldrich (St. Louis, MO, USA), and MβCD was dissolved in 30% D_2_O. 4XQ and 4XL peptides (Fig. 1) were synthesized using Fmoc-based chemistry, and the purity was >90% as confirmed by HPLC (Shimadzu, Kyoto, Japan) and MALDI-TOF-MS (Bruker Japan K.K., Kanagawa, Japan). The peptides were dissolved in ethanol and the concentrations were determined by absorbance using the extinction coefficient of tryptophan.

### Lipid vesicle preparation

POPC vesicles (H-vesicles) and *d*_31_-POPC vesicles (D-vesicles) were prepared as previously reported.^15^ The lipid film was solvated with Tris buffer (10 mM Tris, 150 mM NaCl, 1 mM EDTA, 0.01% NaN_3_) containing 30% D_2_O. The lipid suspension was extruded using a 100 nm-pore polycarbonate filter, and the diameter of the obtained particles was confirmed to be ∼120 nm by dynamic light scattering (FPAR-1000 particle analyzer, Otsuka Electronics, Osaka, Japan). Lipid concentrations were determined using an enzymatic assay kit for choline (Wako, Osaka, Japan).

### Time-resolved small-angle neutron scattering (SANS)

SANS measurements were carried out using TAIKAN at the Material and Life Science Experimental Facility (MLF) of the Japan Proton Accelerator Research Complex (J-PARC), Tokai, Japan, as previously described.^15^ Samples were measured in quartz cells with a pass length of 1 mm. Equal amounts of H-vesicles and D-vesicles were mixed before the addition of the peptide solution (final ethanol concentration: 2.0% v/v). After incubation at 37 °C for 5 min to insert the peptide into the vesicles, MβCD was added and time-resolved measurements were started immediately, where the final concentrations of lipids and MβCD were 30 and 1 mM, respectively. The *I*(*q*) between 0.007 < *q *<* *0.12 was integrated as *I*(*t*). The normalized contrast was obtained from the following equation:^13,14^

$$\frac{\Delta \rho \left(t\right)}{\Delta \rho \left(0\right)}=\frac{\sqrt{I\left(t\right)}-\sqrt{I\left(\infty \right)}}{\sqrt{I\left(0\right)}-\sqrt{I\left(\infty \right)}},$$ (1)

where *I*(0) is the intensity of the mixture of D- and H-vesicles before the addition of MβCD, and *I*(∞) is the intensity of vesicles after completing lipid exchange by MβCD and the peptides (*4SQ* and *4EQ*).

## RESULTS AND DISCUSSION

### Data analysis of SANS measurements

**FIG. 2.**
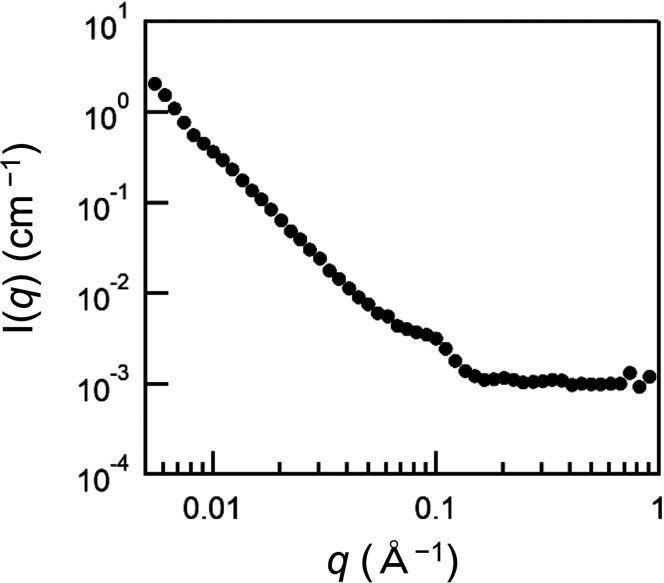
SANS profile of the equivalent mixture (15 + 15 mM) of D- and H-vesicles in Tris buffer containing 30% D_2_O without MβCD.

**FIG. 3.**
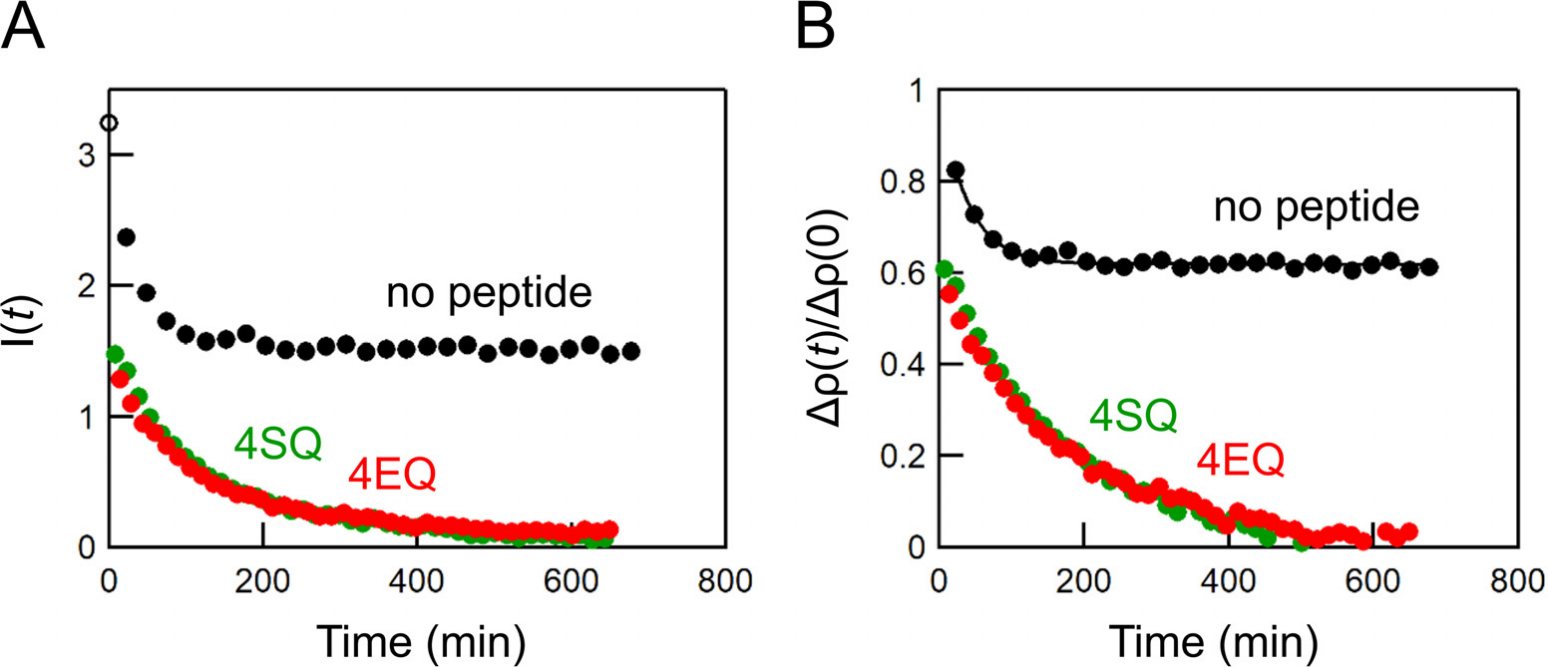
(a) Intensity decay curves of equivalent mixtures of D- and H-vesicles (total 30 mM) without MβCD [*open circle*, which was set to *I*(0)], with 2% EtOH and 1 mM MβCD [*no peptide* (black)], and with 0.03 mol. % peptides and 1 mM MβCD [*4SQ* (green) and *4EQ* (red)]. Under the *no peptide* condition, time-resolved SANS measurement was started after the addition of MβCD. Under the *4SQ* and *4EQ* conditions, the outer leaflet lipids were first completely exchanged between vesicles by incubation with MβCD in the absence of peptides for approximately 600 min, and then the time-resolved measurement was started after the addition of peptides. The average of the intensity at the last points (*t *=* *643 and 649 min, respectively) in the *I*(*t*) profile of *4SQ* and *4EQ* was set to *I*(∞). (B) Normalized contrast profiles calculated from Eq. (1). The solid line is a fitting curve according to Eq. (9), where *k*_f_ was fixed to 0.

The SANS profile of the mixture of D- and H-vesicles provided a small peak at *q* ∼ 0.1 Å^−1^, corresponding to a lamellar repeat distance of ∼60 Å (Fig. 2),^16^ which indicates the presence of multilamellar vesicles. Therefore, it is necessary to change the previously derived equation [Eq. (S1) in the supplementary material] used for the analysis of time-resolved SANS data of large unilamellar vesicles.^13^ In the time-resolved SANS experiments, MβCD and lipid scrambling peptides were added to the pre-formed vesicles, so that intervesicular lipid exchange by MβCD and flip-flop by the peptides would occur only in the outermost bilayer. Therefore, we speculated that setting the intensity of the vesicles after completion of the lipid exchange by MβCD and peptides to *I*(∞) could eliminate the contribution of the inner layers of multilamellar vesicles to the contrast. The calculated normalized contrast decay curve for vesicles with MβCD, however, did not reach 0.5, only reaching ∼0.6, and the addition of the scrambling peptides further reduced the contrast from 0.6 to almost zero (Fig. 3). The difference in the contrast reduction before and after peptide addition (approximately 0.4 and 0.6, respectively) suggests that lipid exchange occurred even in the internal bilayer of multilamellar vesicles, presumably due to the close contact of the bilayers. In other words, the contrast decay after peptide addition is due to the lipid flip-flop in the outermost bilayer and the lipid exchange between the outermost bilayer and the secondary bilayer. Considering that the added peptides are thought to be inserted only in the outermost bilayer of the multilamellar vesicles and that spontaneous POPC flip-flop is negligible on our experimental time scale (Fig. 3), flip-flop in the secondary bilayer can be ignored. Therefore, the normalized contrast can be described as follows:

$$\frac{\Delta \rho \left(t\right)}{\Delta \rho \left(0\right)}=C\left(\left|\Delta \rho_{\mathrm{out},1}(t)\right|+\left|\Delta \rho_{\mathrm{in},1}(t)\right|\right)+\left(1-2C\right)\left|\Delta \rho_{\mathrm{out},2}(t)\right|,$$ (2)

where |Δρ_out,1_(*t*)|, |Δρ_in,1_(*t*)|, and |Δρ_out,2_(*t*)| are the contrasts of the outer and inner leaflets of the outermost bilayer and the outer leaflet of the secondary bilayer, respectively, and (1–2*C*) represents the fraction of the outer leaflet of the secondary bilayer. The kinetics of lipid exchange in each leaflet is described by the following differential equations with the rate constants of the MβCD-mediated exchange (*k*_ex_), flip-flop (*k*_f_), and exchange between the outermost bilayer and the secondary bilayer (*k*_ex,MLV_)

$$-\frac{d\left|\Delta \rho_{\mathrm{out},1}\right|}{dt}=k_{\mathrm{ex}}\left|\Delta \rho_{\mathrm{out},1}\left(t\right)\right|+k_{f}\left(\left|\Delta \rho_{\mathrm{out},1}\left(t\right)\right|-\left|\Delta \rho_{\mathrm{in},1}\left(t\right)\right|\right),$$ (3)

$$-\frac{d\left|\Delta \rho_{\mathrm{in},1}\right|}{dt}=-k_{f}\left(\left|\Delta \rho_{\mathrm{out},1}\left(t\right)\right|-\left|\Delta \rho_{\mathrm{in},1}\left(t\right)\right|\right)+k_{\mathrm{ex},\mathrm{MLV}}\left(\left|\Delta \rho_{\mathrm{in},1}\left(t\right)\right|-\left|\Delta \rho_{\mathrm{out},2}\left(t\right)\right|\right),$$ (4)

$$-\frac{d\left|\Delta \rho_{\mathrm{out},2}\right|}{dt}=-\frac{C}{1-2C}k_{\mathrm{ex},\mathrm{MLV}}\left(\left|\Delta \rho_{\mathrm{in},1}\left(t\right)\right|-\left|\Delta \rho_{\mathrm{out},2}\left(t\right)\right|\right).$$ (5)

However, it is hardly possible to analytically derive the solution of these differential equations, which would be a triple exponential function. Moreover, it is difficult to determine the kinetic parameters unambiguously by fitting the contrast decay profile with a triple exponential function.

If the lipid exchange between the outermost and secondary bilayers is sufficiently faster than the flip-flop, then |Δρ_in,1_(*t*)| can be considered equal to |Δρ_out,2_(*t*)|, and the following equations can be applied instead of Eqs. (2)–(5):

$$\frac{\Delta \rho \left(t\right)}{\Delta \rho \left(0\right)}=C\left|\Delta \rho_{\mathrm{out},1}(t)\right|+\left(1-C\right)\left|\Delta \rho_{\mathrm{in},1}(t)\right|,$$ (6)

$$-\frac{d\left|\Delta \rho_{\mathrm{out},1}\right|}{dt}=k_{\mathrm{ex}}\left|\Delta \rho_{\mathrm{out},1}\left(t\right)\right|+k_{f}\left(\left|\Delta \rho_{\mathrm{out},1}\left(t\right)\right|-\left|\Delta \rho_{\mathrm{in},1}\left(t\right)\right|\right),$$ (7)

$$-\frac{d\left|\Delta \rho_{\mathrm{in},1}\right|}{dt}=\frac{-C}{1-C}k_{f}\left(\left|\Delta \rho_{\mathrm{out},1}\left(t\right)\right|-\left|\Delta \rho_{\mathrm{in},1}\left(t\right)\right|\right).$$ (8)

Solving the above differential equations under the initial condition of |Δρ_out,1_(0)| = |Δρ_in,1_(0)| = 1, we obtained the final form of the normalized contrast

$$\begin{array}{c} \frac{\Delta \rho \left(t\right)}{\Delta \rho \left(0\right)}=\left\{\frac{1}{2}-\frac{\left(1-2C\right)k_{\mathrm{ex}}+k_{f}/(1-C)}{2X}\right\}\exp\left(-\frac{k_{\mathrm{ex}}+k_{f}/(1-C)+X}{2}t\right)\;\\ +\left\{\frac{1}{2}+\frac{\left(1-2C\right)k_{\mathrm{ex}}+k_{f}/(1-C)}{2X}\right\}\exp\left(-\frac{k_{\mathrm{ex}}+\frac{k_{f}}{1-C}-X}{2}t\right), \end{array}$$ (9)

where

$$X=\sqrt{{k_{\mathrm{ex}}}^{2}+\frac{2\left(1-2C\right)}{\left(1-C\right)}k_{\mathrm{ex}}k_{f}+\frac{1}{{\left(1-C\right)}^{2}}{k_{f}}^{2}}.$$ (10)

To reduce the number of variables in the flip-flop analysis, *C* was fixed to the value obtained from the fitting by Eq. (9) in Fig. 3 (*C* = 0.382).

### Time-resolved SANS

Before evaluating the scramblase activity of the peptides, we assessed the effect of ethanol on spontaneous POPC flip-flop because all peptides used in this study were dissolved in ethanol. The contrast decay profile of the MβCD-mediated lipid transfer in the presence of 2.0% ethanol (Fig. 3, *k*_ex_ = 2.59 × 10^−2 ^min^−1^) was identical to that of our previous results without ethanol,^14^ indicating that 2.0% ethanol did not affect either the POPC flip-flop or the MβCD-mediated lipid transfer. We therefore fixed the concentration of ethanol at 2.0% in all following experiments. Figure 3 also shows that the normalized contrasts were decreased without a delay by addition of the scrambling peptides to the mixed vesicles equilibrated with MβCD, suggesting that the peptides were inserted into the vesicles immediately.

**FIG. 4.**
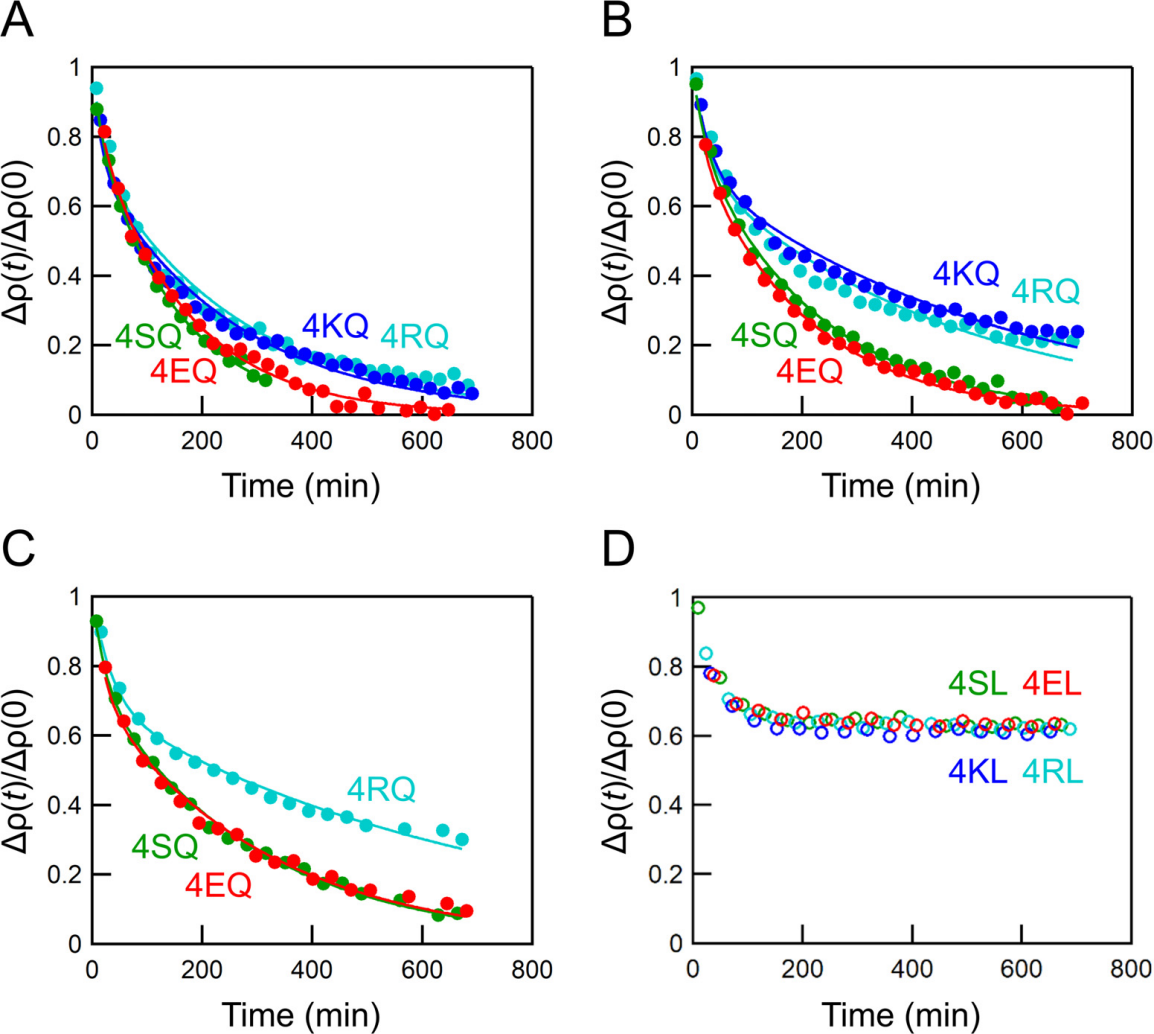
Normalized contrast decay curves of the equivalent mixture of D- and H-vesicles (total 30 mM) with 0.03 mol. % (a), 0.015 mol. % (b), or 0.0075 mol. % 4XQ peptides (c), or 0.03 mol. % 4XL peptides (d) after the addition of MβCD. Solid lines are fitting curves according to Eq. (9).

To evaluate their scramblase activity, peptides were added to the vesicles beforehand and the time-resolved measurements were started with the addition of MβCD. The contrast decay profiles of vesicles with various inserted peptides are shown in Fig. 4 or Fig. S1 in the supplementary material, where the profiles are fitted with either Eq. (9) or Eq. (S1), respectively. Fitting using Eq. (9) was better than that of Eq. (S1), suggesting that the POPC exchange occurs between the outermost and secondary bilayers in the timescale of the experiments. Here, we assumed that lipid exchange between the proximal bilayers in multilamellar vesicles is sufficiently faster than the flip-flop to simplify the calculation of the differential equations. Although spontaneous interbilayer exchange of POPC has been reported to be slow,^14^ Wu and Lentz observed rapid lipid exchange when the bilayers were brought into close proximity using 10% polyethylene glycol (PEG).^17^ Indeed, the rate of POPC inter-vesicular exchange induced by 10% PEG was comparable to that induced by 1 mM MβCD (unpublished data). In the presence of 10% PEG, the fluid space between the aggregated vesicles is approximately 10 Å, as revealed by x-ray diffraction,^18^ and the fluid space between the proximal bilayers in our multilamellar vesicles, calculated based on the SANS profile (Fig. 2), also corresponds to this distance. Based on these facts, it is suggested that a fast lipid exchange between the proximal bilayers occurs. Therefore, Eq. (9) was used in the subsequent analysis, although this is a semi-quantitative consideration that does not accurately account for this lipid exchange rate.

A rapid flip-flop (*k*_f_ = 7.37 × 10^−3 ^min^−1^) was observed in vesicles containing 0.03 mol. % 4KQ, which has almost the same sequence as the scrambling peptide TMP25Q, except for the position of the terminal lysine residues, suggesting that 4KQ has a transmembrane conformation in the membrane and exhibits equivalent scramblase activity [Fig. 4(a)]. Conversely, the addition of 4KL did not induce the POPC flip-flop [Fig. 4(d)]. These results indicate that the central glutamine residue is critical for the scramblase activity on the natural occurring lipid (POPC), which is consistent with our previous results using a fluorescence technique,^12^ and that the peptide insertion itself does not perturb the membrane.

The four N-terminal lysine residues of 4KQ and 4KL were substituted with other hydrophilic residues (arginine, glutamic acid, or serine) to evaluate the effect of the anchoring residues on the POPC flip-flop. All 4XQ peptides accelerated the flip-flop of POPC. At a peptide concentration of 0.03 mol. %, 4RQ showed scramblase activity (*k*_f_ = 7.24 × 10^−3 ^min^−1^) comparable to that of 4KQ, whereas 4EQ and 4SQ showed higher activity (1.47 × 10^−2^ and 1.60 × 10^−2 ^min^−1^, respectively). In contrast, no scramblase activity was observed in vesicles with 4RL, 4EL, or 4SL [Figs. 4(a) and 4(d)], again suggesting the importance of the central glutamine residue.

**FIG. 5.**
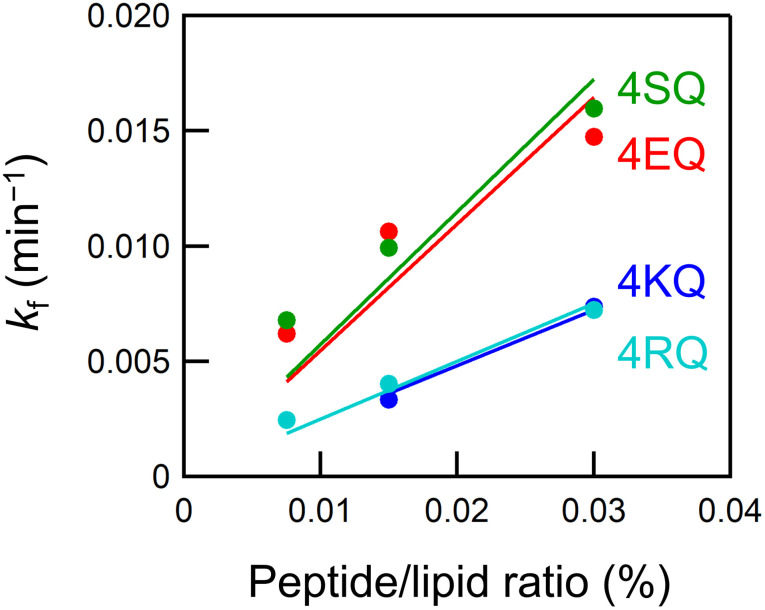
Flip-flop rate constants in vesicles containing 0.0075–0.03 mol. % 4XQ peptides. Solid lines represent the fitted linear regression curve.

To gain more insight into the lipid scrambling mechanisms of the peptides, we assessed the peptide concentration dependence of the flip-flop rate [Figs. 4(a)–4(c)]. The flip-flop rates in vesicles containing the scrambling peptides increased in proportion to the peptide/lipid ratio, suggesting that the peptides promote the flip-flop in their monomeric form (Fig. 5). Therefore, the lipid scrambling by 4XQ peptides might be due to membrane perturbation by the exposure of a hydrophilic glutamine residue to the hydrophobic region of the membrane, as we have previously proposed.^12^ This is quite different from the toroidal pore mechanism of amphiphilic peptides.^8^ The scramblase activities per peptide monomer were calculated from the slope of the concentration dependence, and are listed in Table I. The activities of 4XQ peptides were similar to those of our previously developed scrambling peptides, which were evaluated using fluorescently labeled lipids.^11^ 4SQ and 4EQ had approximately twofold higher activities than those of 4KQ and 4RQ. We previously revealed that a scrambling peptide with a lysine residue in the center of the sequence has lower scramblase activity than one with a glutamic acid residue.^10^ The energy barrier for phospholipid flip-flop is the translocation of the hydrophilic headgroup through the hydrophobic hydrocarbon region. PC is a zwitterionic lipid, and the repulsive electrostatic interaction between its quaternary ammonium group and a lysine or arginine residue might be unfavorable for the PC flip-flop because, unlike the phosphate group, the quaternary ammonium group is charged during the flip-flop.

**TABLE I.** Scramblase activities of the 4XQ peptides.

Peptide	Number of lipids translocated by a single peptide (s^−1^)
4KQ	0.40 ± 0.02
4RQ	0.42 ± 0.02
4EQ	0.91 ± 0.12
4SQ	0.96 ± 0.11

## CONCLUSIONS

In this study, we successfully developed membrane-insertable scrambling peptides and evaluated their scramblase activity using semi-quantitative model fitting of time-resolved SANS data. All 4XQ peptides exhibited scramblase activity after addition to the membrane, whereas 4XL peptides had no effect on the POPC flip-flop. The activities of the scrambling peptides increased in proportion to the peptide/lipid ratio, suggesting that the peptide monomers promote the flip-flop. Exposure of the glutamine residue to the hydrocarbon region might perturb the membrane and promote the flip-flop. In addition, terminal hydrophilic residues also mediated peptide scramblase activity; peptides with negatively charged or uncharged polar residues were more effective for the POPC flip-flop. Addition of the scrambling peptides to the membrane readily enhanced the phospholipid flip-flop, and therefore, the peptides could be a valuable tool for manipulating the flip-flop in living cells.

## SUPPLEMENTARY MATERIAL

See the supplementary material for the fitting of time-resolved SANS data using a conventional equation for the analysis of large unilamellar vesicles.
The authors declare no competing financial interest.

## Data Availability

The data that support the findings of this study are available from the corresponding author upon reasonable request.
